# mHealth smoking cessation intervention among high-school pupils (NEXit Junior): study protocol for a randomized controlled trial

**DOI:** 10.1186/s13063-018-3028-2

**Published:** 2018-11-22

**Authors:** Kristin Thomas, Marcus Bendtsen, Catharina Linderoth, Ulrika Müssener

**Affiliations:** Department of Medical and Health Sciences, Faculty of Medicine and Health, Linköping University, Linköping, Sweden

**Keywords:** Tobacco consumption, Smoking cessation, High-school pupils, mHealth intervention, Randomized controlled trial

## Abstract

**Background:**

Mobile health (mHealth) interventions, using text messages to support high-school pupils to quit smoking, could be a novel and cost-effective approach. However, more research is needed, specifically to investigate long-term effectiveness. The study aims to evaluate the effectiveness of a newly developed mHealth intervention targeting smoking cessation among high-school pupils.

**Methods:**

The study is a two-arm, randomized controlled trial with an intervention group (mHealth intervention) and a control group (treatment as usual: national smoking cessation help line). Outcome measures will be investigated at baseline and at 3, 6, and 12 months’ follow-up. Primary outcome measures will be: prolonged abstinence and 4-week point prevalence of smoking abstinence. Secondary outcome measures will be: 7-day point prevalence of smoking abstinence; mean number of quit attempts since taking part in the study; number of uses of other smoking cessation services since first invitation to the study and number of cigarettes smoked weekly if still smoking.

**Discussion:**

High schools in Sweden are bound by law to offer a smoke-free environment. However, little effort has been made to offer support to pupils who wish to quit smoking; rather the emphasis is on prevention of uptake. The study will examine the effectiveness of a stand-alone mHealth intervention targeting smokers among high-school pupils.

**Trial registration:**

The trial was not retrospectively registered and has been registered at ISRCTN with the unique identification number ISRCTN15396225. The trial was registered on 13 October 2017.

**Electronic supplementary material:**

The online version of this article (10.1186/s13063-018-3028-2) contains supplementary material, which is available to authorized users.

## Background

Smoking is responsible for about 10% of the total disease burden in Sweden, and around 6000 people die every year due to smoking [1]. In Sweden, the prevalence of daily smoking among young people aged between 16 and 29 years has been stable during the last 5 years at approximately 11–13% among women and 7–10% among men. The figures for occasional smoking were approximately 19–22% for women and 22–24% for men during the same period [2, 3]. Around 25% of high-school pupils were smoking in 2016 and a third of those were daily smokers [4].

However, most tobacco control programs in schools focus on prevention of uptake rather than offering support for quitting smoking [5]. Implementation of smoking cessation interventions at the high-school level could, therefore, have a major impact on population health. A recent Cochrane review including 28 trials suggested that interventions that have shown to be effective among adults, e.g., motivational enhancement, could also be effective among adolescents. However, the review also showed that there is currently insufficient evidence to recommend a specific method of intervention for young people and that more data are needed on long-term cessation [6]. Furthermore, although recent figures show that around two thirds of high-school smokers want to quit [7], only about one in ten seeks or gains access to evidence-based interventions [8, 9].

During the last decade, the number of Internet and computerized interventions has expanded substantially, moving interventions from the health care sector to the homes of the general population. However, a meta-review, including 41 studies on online prevention aimed at lifestyle behaviors, showed that the majority of users were female, highly educated, white, and living in high-income countries. Also that the overall use of the interventions was low [10].

In contrast to Internet-based interventions delivered on computers, mobile health (mHealth) interventions have the capacity to interact in a more dynamic way as individual messages are delivered at certain predetermined intervals. Text-based interventions also offer other advantages. Users can access text-messaging services (SMS) whenever they feel an urge to smoke, and these interventions can be provided to individuals within their own environment and delivered in real time [11–14]. Also, mHealth interventions using a “push” technique, such as text messages, do not require the user to seek out information or maintain engagement by going to a website. As the growth of mobile phone ownership and usage is expected to continue to grow, mobile technology offers an innovative way to reach smokers worldwide [14].

Various challenges and tasks can be sent via text messages that require a user response. This may allow two-way communication in real-time, facilitating adherence to the intervention and providing short, timed bursts of information throughout the day. Because nearly all young people have a mobile phone, mHealth interventions could reach a large proportion of the target group and could have a significant impact as a result of the high levels of exposure, low cost, and strong adherence to text-based interventions [15, 16].

There is increasing evidence that text-messaging programs on mobile phones can help people to modify their health behaviors. The evidence is particularly strong for smoking cessation. A recent Cochrane review indicated that smoking cessation programs by text messaging increased long-term quit rates by 67% [15]. Text messages have been found to help increase access to educational and support services that promote smoking cessation across diverse populations [14]. A recent meta-review on text-message interventions for smoking cessation, including 13 studies, showed that cessation rates for the intervention group were 36% higher than for the control group [17]. An earlier review including five studies showed a similar short-term effect of text-message interventions on smoking cessation; however, limited long-term effects were found [17]. Interventions including text messages have also been found to be effective among young adults [11, 16]. A study aiming to test the efficacy of a text-based intervention in high school showed significant smoking abstinence among occasional smokers [18]. However, the study included a small sample size. A meta-analysis investigating text-messaging interventions targeting smoking among adolescents concluded that although there is supporting evidence, a greater understanding is needed on how the interventions work, i.e., mechanisms of change. Furthermore, to our knowledge, no studies have been performed in Sweden targeting high-school pupils, and there is a need for an effective intervention that can be implemented and offered via high schools.

In our previous research, we developed an mHealth intervention, NEXit, targeting smoking among university students. NEXit was developed by our research group, using formative research methods (for more details [19]). The text messages in the main NEXit study were developed based on existing evidence-based practice, expert guidance, and official smoking cessation manuals recommended in Sweden. Key elements from previous text- and Internet-based interventions were also included, e.g., problem-solving tips and distraction techniques. The effectiveness of the main NEXit intervention was evaluated in a two-arm randomized controlled trial (RCT) including 1590 participants of between 21 and 30 years of age. The trial showed a significant difference between the intervention group (25.9%) and the control group (delayed intervention) (14.6%) on 8-week prolonged abstinence. For 4-week point prevalence of complete cessation, the numbers were 20.6 and 14.2%, respectively. Students who received the intervention were almost twice as likely to quit smoking than students in the control group [20].

This article presents the study protocol of a RCT testing the effectiveness of an mHealth intervention targeting smoking among high-school pupils in Sweden.

## Methods

### Design

The study is a two-arm RCT with an intervention group (mHealth smoking cessation program) and a control group (treatment as usual: national smoking cessation help line). Equal number of individuals will be allocated in the two groups.

### Aim and hypotheses

The study aims to evaluate the effectiveness of a newly developed mHealth intervention targeting smoking among high-school pupils. The primary hypothesis is that participants in the intervention group will report significantly higher cessation rates (measured by the primary outcome measures) at follow-up compared with participants in the control group. Secondary hypotheses are that the intervention group will report significantly higher rates of 7-day point prevalence of smoking abstinence, higher mean number of quit attempts since taking part in the study, fewer number of uses of other smoking cessation services since first invitation to the study, and lower numbers of cigarettes smoked weekly if still smoking at follow-up, compared with the control group. These hypotheses are proposed at 3, 6, and 12 months’ follow-up.

### Outcome measures

Outcome measures will be investigated via questionnaires at baseline and at 3, 6, and 12 months after enrollment. The primary outcomes will be: (1) prolonged abstinence (not smoked more than five cigarettes in the last 8 weeks) and (2) 4-week point prevalence of smoking abstinence. Secondary outcomes will be: (3) 7-day point prevalence of smoking abstinence; (4) mean number of quit attempts since taking part in the study; (5) number of uses of other smoking cessation services since first invitation to the study, e.g., calling help line; and (6) number of cigarettes smoked weekly if still smoking.

### Intervention group

The intervention will consist of a 12-week automated mHealth program with a total of 121 text messages. The program starts with two to four messages per day the first 2 weeks. In week 3, the participants will receive two messages per day. The number of messages per day will be reduced to one during weeks 4–7 and in weeks 8–12, a total of five to six messages per week. The intervention is based on a similar program developed for an adult target group [20]. The intervention was developed using formative methods, including focus groups with university students, an expert panel with university students and professionals, and behavior-change technique analysis [21]. The content of the text messages will include information about the health risks of smoking, tips on behavior-change strategies, and activities. The intervention included the following elements: making a public declaration about quitting, encouraging asking for support from family and friends, distraction techniques and tips to avoid weight gain, tips to cope with cravings and to avoid smoking triggers, and how to distract one’s mind from smoking.

### Control group

The control group will be offered treatment as usual. High-school pupils in Sweden can turn to school-based health centers, including nurses and counselors. The school health centers do offer smoking cessation support. Participants in the control group will be informed via a text message that they have been allocated to the control group. The text message will include information on where they can gain smoking cessation support, i.e., the national smoking cessation help line offering smoking cessation support to youth. No additional prompts, reminders, or information will be given to the control group during the study.

### Randomization

Participants will be randomized into two groups; one group will receive the novel intervention and the other will be referred to treatment as usual. Each participant will be allocated a number (1 or 2) with equal probability using Java’s built-in random number generator (java.util.Random). Randomization is thus fully computerized, does not use any strata or blocks, and cannot be subverted, because this and all subsequent study processes are fully automated. A runs test will be performed to test if the final allocation sequence can be considered to have been generated from a random process. The participants will know that they have been randomized to either the intervention group or the control group.

The risk of contamination between the intervention and control group is present in almost all trials of eHealth interventions as information is easily shared among participants and no restrictions can be made to make certain that only the intervention group has access to the intervention. In some cases cluster randomization may reduce the risk; however, it may also induce overconfidence that the risk has been entirely removed. Pupils attending high school in Sweden are for administrative reasons subdivided into something that resembles a traditional school class; however, pupils are mixed across classes and schools when attending courses. There is, therefore, no logical unit that shields or reduces interaction among pupils. Furthermore, with the growth of interaction among young adults using social platforms there is no longer a geographical limitation that could be used for clustering. Clustering would, therefore, in this case only accomplish a false sense of bias reduction. Analysis by treatment allocated disregarding potential contamination may lead to underestimation and, therefore, we will conduct a sensitivity analysis taking into account hypothesized probabilities of contamination rates at 5, 10, 15 and 20% (i.e., the probability that a control group participant had access to the intervention).

### Participants and procedure

**Fig. 1:**
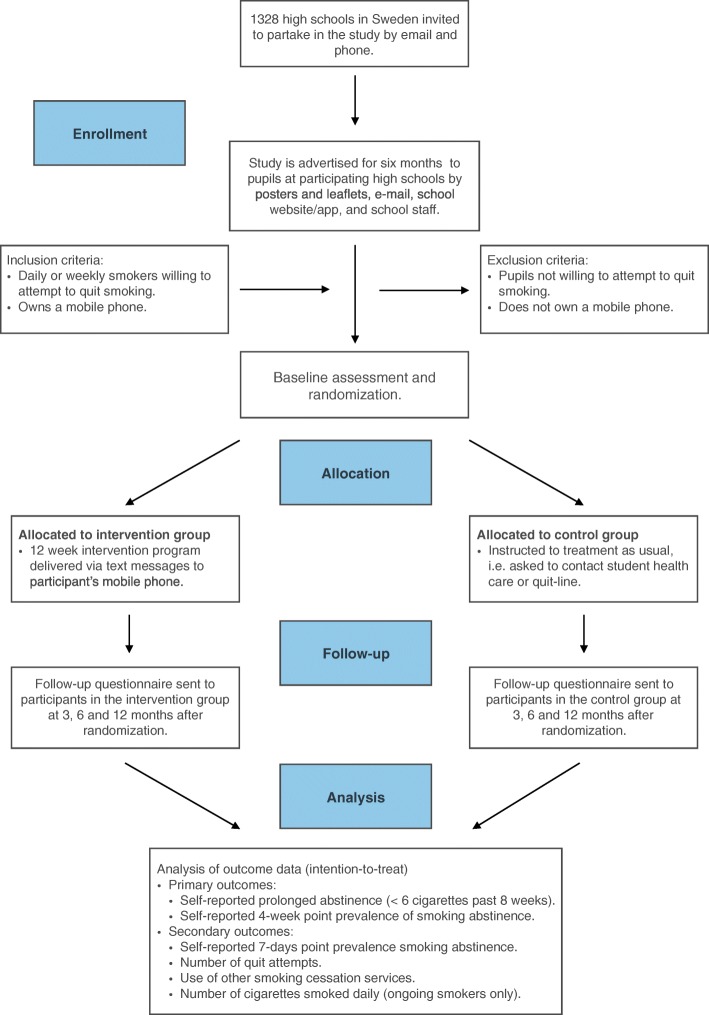
Consolidated Standards of Reporting Trials (CONSORT) flowchart

**Fig. 2:**
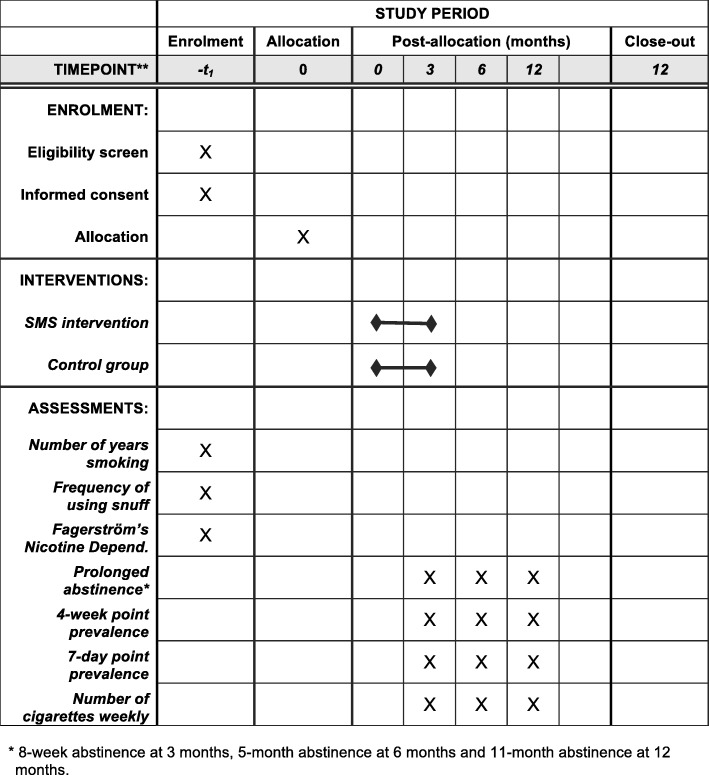
Standard Protocol Items: Recommendations for Interventional Trials (SPIRIT) Figure

Participants will be recruited through advertisements in high schools in Sweden. High school in Sweden is a free and voluntary level of education after primary school. The majority of pupils attending high school in Sweden are between 16 and 19 years of age. Inclusion criteria will be high-school pupils who are daily or weekly smokers, are willing to attempt to quit smoking, and own a mobile phone. A flowchart of the recruitment procedure for the study is presented in Fig. 1 and a Standard Protocol Items: Recommendations for Interventional Trials (SPIRIT) Figure is presented in Fig. 2.

School principals will be contacted via e-mail and invited to take part in the study. Recruitment will last for 6 months and be executed by paper advertising (posters and leaflets), digital advertising (e-mail, school website, or app if applicable), and by school staff (teachers, mentors, and/or school health centers). Pupils will register their interest by sending a text message to a dedicated telephone number (included in all information materials). Informed consent and the baseline questionnaire will be completed on their mobile phone. After completing the baseline questionnaire, participants will immediately be randomized to the intervention group or the control group. A text message will be sent to each participant to inform them to which group they have allocated.

All participants will be sent a follow-up questionnaire via a text message (with a link to the questionnaire) at 3, 6, and 12 months after enrollment (3-month interval will be primary outcome). Three reminders, 2 days apart, will be sent to non-responders, also via text message. In addition, participants who still do not respond will be telephoned every second day, with a maximum of three phone calls. These phone calls will only include two questions investigating the primary outcomes (prolonged abstinence and 4-week point prevalence of smoking abstinence).

### Baseline questionnaire

The baseline questionnaire will include a total of 14 items investigating (1) number of year(s) of smoking, (2) frequency of using snuff, (3–8) Fagerström’s Nicotine Dependence Scale, (9) readiness to quit within the next 4 weeks, (10) number of previous quit attempts, (11) previous use of other smoking cessation aids, (12) previous support from smoking cessation specialist, (13) how they found out about the program, and (14) gender.

### Follow-up questionnaire

The follow-up questionnaire will include seven questions investigating the primary and secondary outcomes: (1) prolonged abstinence (not smoked more than five cigarettes in the last 8 weeks at the 3-month follow-up; 5 months at the 6-month follow-up; and 11 months at the 12-month follow-up), (2) 4-week point prevalence of smoking abstinence, (3) 7-day point prevalence of smoking abstinence, (4) numbers of cigarettes smoked weekly if still smoking, (5) mean number of quit attempts since taking part in the study, (6) number of uses of other smoking cessation services since first invitation to the study, e.g., calling help line, and (7) frequency of using snuff.

### Power calculation

The power calculation is based on our earlier research [20], where 25.9% reported prolonged abstinence in the intervention group and 14.6% in the control group, i.e., an 11.3% difference between the two groups. To achieve 80% power with a significance level of 0.05 (two-sided) and correction for continuity, a sample size of 195 persons is needed in each group. If there is 30% attrition in the follow-up measurements, the number needed in each group is 279, with a total required sample size of 558.

### Data analysis

The data analysis will conform to a pre-specified statistical analysis plan. The analyses will start after collection of the 3-month follow-up data. There will be no interim analyses or stopping rules. Following the intention-to-treat analysis strategy, all analyses will include all participants with follow-up data in their groups as randomized, and sensitivity analyses will include all randomized participants to explore different assumptions about any missing data. Descriptive analyses will include summary tables (descriptive statistics and/or frequency tables) for baseline and follow-up variables as appropriate. A flowchart of recruitment of participants will be generated, including, e.g., the number of students screened and the number included in the primary and secondary analyses.

The binary outcomes of self-reported prolonged abstinence, 4-week prevalence of complete smoking cessation, and 7-day point prevalence of smoking abstinence will be analyzed by logistic regression and the results presented as odds ratios and 95% confidence intervals (CIs). Number of quit attempts and number of uses of other smoking cessation services will be analyzed by negative binomial regression and the results presented as ratios of means (95% CI). Number of cigarettes smoked weekly will be analyzed by logarithmic transformation and linear regression and the results presented as the ratio of geometric means (95% CI). All regression analyses will be adjusted for the following baseline variables: gender, duration of smoking, average number of cigarettes smoked weekly, severity of dependence as measured by Fagerström’s Nicotine Dependence Scale, and amount of snuff used at baseline. Effect modification analyses will be performed for the two primary outcomes and the following potential effect modifiers measured at baseline: gender, average number of cigarettes smoked weekly, amount of snuff used weekly, and severity of dependence. Each effect modification analysis will be performed by comparing adjusted logistic regression models, excluding and including the interaction parameter using the likelihood ratio test.

A sensitivity analysis will explore the effects of departures from the missing-at-random (MAR) assumption in the main analysis. We will use data on the number of follow-up texts and phone calls needed before an individual responded to explore the plausibility of the MAR assumption by exploring the association between quitting and the number of follow-up attempts needed.

All tests will be performed two-sided with a 5% level of significance.

## Discussion

All high schools in Sweden are bound by law to offer a smoke-free environment for their pupils. However, little effort has gone into finding effective ways of systematically helping pupils who wish to quit smoking; the emphasis is on the prevention of uptake. Following the success of our NEXit smoking cessation trial among university students, we believe that a similar mHealth intervention targeting high-school pupils may also be effective. The potential direct benefit from the trial is an intervention program that can be made available nationwide at very low cost, requiring little or no resources from participating high schools.

Although smoking cessation programs delivered via text messages have been evaluated previously, there has not been much research on the medium- and long-term effects of the intervention. The current research project, therefore, investigates not only the immediate effect at 3 months but also the intermediate and long-term effects at 6 and 12 months.

A limitation of the study is that follow-up will not be biochemically verified, but will rather rely on participants’ self-reported abstinence. However, any bias in reporting may be assumed to be equal in both the intervention group and the control group. Also, The Society for Research on Nicotine and Tobacco recommends that, in population-based studies with limited face-to-face contact, it is neither required nor desirable to use biochemical verification [22].

### Trial status

At the time of submission, the trial had not started. An invitation to all high schools in Sweden had been sent with a request to partake in the study. This report describes the protocol, version 2.0 2018-01-19, and adheres to the Standard Protocol Items: Recommendations for Interventional Trials (SPIRIT) reporting guidelines (see Additional file 1)).

## Additional file

Additional file 1: Standard Protocol Items: Recommendations for Interventional Trials (SPIRIT) Checklist. (PDF 71 kb)

## Abbreviations

CI: Confidence interval
CONSORT: Consolidated Standards of Reporting Trials
MAR: Missing at random
NEXit: Nicotine EXit, the name of the intervention
RCT: Randomized controlled trial
SMS: Short message service, more popularly called text messages
